# The Impact of Mood Disorders on Adherence, on Life Satisfaction and Acceptance of Illness—Cross-Sectional Observational Study

**DOI:** 10.3390/healthcare12232484

**Published:** 2024-12-09

**Authors:** Alicja Jeżuchowska, Anna Maria Cybulska, Kamila Rachubińska, Karolina Skonieczna-Żydecka, Artur Reginia, Mariusz Panczyk, Dorota Ćwiek, Elżbieta Grochans, Daria Schneider-Matyka

**Affiliations:** 1 Department of Clinical Nursing, Wroclaw Medical University, 51-618 Wroclaw, Poland; alicja.jezuchowska@umw.edu.pl; 2 Department of Nursing, Pomeranian Medical University in Szczecin, 71-210 Szczecin, Poland; anna.cybulska@pum.edu.pl (A.M.C.); kamila.rachubinska@pum.edu.pl (K.R.);; 3 Department of Biochemical Science, Pomeranian Medical University in Szczecin, Broniewskiego 24, 71-460 Szczecin, Poland; 4 Department of Psychiatry, Pomeranian Medical University in Szczecin, 71-460 Szczecin, Poland; areginia@gmail.com; 5 Department of Education and Research in Health Sciences, Medical University of Warsaw, 14/16 Litewska St., 00-581 Warsaw, Poland; mariusz.panczyk@wum.edu.pl; 6 Department of Obstetrics and Pathology of Pregnancy, Pomeranian Medical University in Szczecin, 71-210 Szczecin, Poland; dorota.cwiek@pum.edu.pl

**Keywords:** adherence, life satisfaction, acceptance of illness, mood disorders, mental

## Abstract

**Background:** Mood disorders are among the most prevalent and debilitating mental conditions in worldwide populations. The aim of this study was to identify the factors influencing life satisfaction, disease acceptance, and therapeutic adherence among people with mood disorders. **Methods**: This survey-based study included 103 people with mood disorders. It was performed using the author questionnaire, and standardized research tools, namely the Adherence to Refills and Medication Scale (ARMS), the Acceptance of Illness Scale (AIS), the Beck Depression Inventory (BDI), and the Satisfaction with Life Scale (SWLS). **Results:** The level of life satisfaction decreased with the increase in the severity of the depressive symptoms (*SE* = −0.665, *p* < 0.001). Mood disorder patients with more severe depressive symptoms had significantly higher scores on the adherence scale (*SE* = 0.290, *p* = 0.003). The patients with higher levels of depressive symptoms showed a lower level of acceptance of the disease. **Conclusions:** 1. The dosage of medications taken, and the severity of the depressive symptoms determine life satisfaction of people with mood disorders. 2. The respondents with a greater severity of depressive symptoms scored higher on the adherence scale, which means that they were more likely to be non-adherent to the treatment recommendations. The type of mood disorder may affect patient adherence. The subjects with bipolar disorder showed higher adherence and those with anxiety–depressive disorder showed a lower adherence than the patients with depression. 3. The subjects with more severe depressive symptoms showed a lower degree of acceptance of the disease.

## 1. Introduction

A low level of bad mood, persisting regardless of external and internal factors, is a sign of mental well-being and a determinant of an individual’s health [1]. In clinical practice, mood changes are axial symptoms of affective disorders, the two most important of which are depressive disorder and bipolar affective disorder. Mood disorders can also occur in the course of psychosis, and are then accompanied by psychopathological symptoms, such as delusions, disorganized thoughts, and hallucinations. Mood changes, especially mood decline, are also associated with anxiety and personality disorders [2,3].

The occurrence of mental disorders influences previous functioning, quality of life, and life satisfaction [4]. Mood disorders are among the most prevalent and debilitating mental conditions in worldwide populations. They are the second most common category of psychiatric problems (just after anxiety disorders), and contribute to increased mortality among those affected [5]. The symptoms of mood disorders are numerous, diverse, and occur with varying frequency. The primary ones include lowered mood, loss of interest, and anhedonia, as well as lower energy or fatigue endurance. Other very common but less specific symptoms are lowered self-esteem, feelings of guilt, feeling worthless, a pessimistic perception of future events, suicidal thoughts or behavior, self-injurious behavior, sleep problems, decreased appetite, and concentration difficulties [6]. Data from the World Health Organization (WHO) show that about 264 million people in the world suffer from depression, and 45 million people have bipolar affective disorder [7,8]. Both conditions contribute to a poor quality of life (QoL) and increased mortality. Depression ranks as the second most common factor leading to the inability to work due to disability, while bipolar affective disorder ranks sixth [9]. According to the EZOP II study (Epidemiology of Psychiatric Disorders and Availability of Psychiatric Health Care) conducted by the Institute of Psychiatry and Neurology, mood disorders affect nearly 1.5 million people, and more than 8 million (26%) have experienced at least one mental disorder in their lives, with only 16% of sufferers receiving specialist help [10]. Both psychosocial and biological factors have an impact on the development of mood disorders in a patient. Stress factors (life events) can largely trigger an episode of illness and the emergence of chronic symptoms. However, it should be taken into account that the etiology of mood disorders has not yet been fully elucidated. Numerous genetic studies are currently being conducted on the influence of genes on the inheritance of the disease [1].

The basis for the proper management of people with mood disorders is to tailor treatment methods to the patient’s condition and diagnosis. Current treatment methods, i.e., antidepressants and psychotherapy, are recommended as first-line treatment. These methods have changed the lives of patients around the world for the better and will probably continue to do so in the coming years [11]. It is also important to pay attention to comorbidities, appropriate drug tolerance, efficacy, side effects and age, and to build an appropriate therapeutic relationship based on cooperation (including the provision of basic information prior to treatment). The goal of therapy for mood disorders is to achieve symptomatic remission and return to premorbid functioning. The determining factor in selecting a drug is not its efficacy alone, but also the patient’s tolerance, as well as its side effects and interactions with other medicines [12].

Non-adherence to therapeutic recommendations is one of the most common clinical problems in primary care, leading to deterioration in a patient’s quality of life, additional financial costs, increased morbidity, and mortality [13,14]. The factors influencing adherence to medical recommendations include cognitive factors (awareness, the extent to which an individual is able to obtain, process, and understand basic health information, and the services needed to make appropriate health decisions) [15]; interpersonal factors (a doctor–patient relationship—patients who feel motivated by the doctor toward self-care, communicate well with him or her, and agree on the care they have received are more likely to be involved in the process of treatment and adhere to recommendations) [15]; factors related to the health care system (limitations resulting from the duration of the visit, a lack of access to care, and long waiting times may be reasons for not starting treatment) [16]; socio-economic factors (lack of understanding and support from the family, and the high price of medicines) [17]. Non-adherence to treatment recommendations may have serious consequences for patients with mood disorders. There are several methods to show whether the patient cooperates with the doctor and follows his recommendations, or whether he or she skips the prescribed doses of their medications. These include objective methods, such as detecting the drug in the blood, urine, or saliva by analysis or special markers [18], pill counting, electronic monitoring, and an electronic record of the filled prescriptions [19]. Subjective methods involve the assessment of the patient, medical staff, and those in the patient’s immediate environment. The most commonly used subjective methods are an interview, filling out a questionnaire, and an assessment by health care professionals and the patient’s relatives [20]. The problem arises when the patient inadvertently skips medications, which makes it difficult to assess adherence. This may be due to the severity of the disease and poorer cognitive function, and sometimes a change in their daily routine [21].

The World Health Organization has defined quality of life as “an individual’s perception of their position in life in the context of the culture and value systems in which they live and in relation to their goals, expectations, standards and interests” [22]. With the emergence of this definition, the tasks of medicine have been broadened so that they do not focus only on the fight against the disease, but assume a holistic approach to the patient and their satisfaction with functioning on an individual and social level. In psychiatry, an awareness of the information provided and its reliability are important. Measuring life satisfaction aims to benefit the patient, brings relief to sufferers, and contributes to the understanding of symptoms. Numerous studies have confirmed that quality of life indicators are significantly related to the type of disease, its phases and severity, as well as adherence, health status indicators, and social support [23]. Testing the quality of life in mental disorders refers to the patient’s entire situation, without focusing solely on their psychopathological symptoms. A greater severity of disease symptoms translates to a low level of quality of life, and thus a low level of life satisfaction for the patients [24].

Factors such as the course and severity of the disease, treatment methods, and prognosis, as well as the individual predisposition, directly affect the patient’s attitude toward the disease. Greater acceptance of the disease means better adaptation to its consequences and resulting limitations, and reduced psychological discomfort. Patients who accept their disease respond better to emerging symptoms [25,26].

Mood disorders determine patients’ mental functioning, quality of life, and ability to perform social and professional roles. Symptoms of these disorders, including a depressed mood, a lack of energy, or suicidal thoughts, lead to the significant deterioration of their psychological well-being. Despite the availability of effective treatments, including pharmacotherapy, adherence to treatment remains an issue that affects the effectiveness of therapy and improvement in the quality of life. In addition, the patient’s attitude toward the disease, including acceptance of the condition, is crucial to the effectiveness of the treatment and their satisfaction with life.

The aim of the study was to analyze the factors related to the treatment and severity of mood disorders on life satisfaction, disease acceptance, and treatment adherence. Understanding these relationships may contribute to better tailoring of therapeutic approaches and improving patients’ quality of life, as well as influence the effectiveness of the treatment in the context of mental illness.

## 2. Materials and Methods

This study involved a group of 103 people with mood disorders, treated at the Department of Psychiatry of the Pomeranian Medical University in Szczecin. It was based on a survey performed using a questionnaire on sociodemographic and medical data, as well as standardized research tools. The research was conducted from December 2020 to January 2022. The study was conducted during outpatient visits. The severity of the symptoms among the patients in the study did not require hospitalization; they received maintenance treatment. The treatment was used to prevent relapse or reduce the severity of the symptoms that occurred despite treatment. The inclusion criteria for the study were as follows: an age over 18 years, consent to participate in the study, a clinically confirmed mood disorder (depression, bipolar affective disorder, mixed depressive, and anxiety disorders), and the completion of the entire set of questionnaires. Failure to meet at least one of the above conditions was a criterion for exclusion from the study. Since one respondent was excluded, a total of 102 questionnaire sets were eventually analyzed. The patients had been diagnosed with mental disorders by a physician, based on the tenth revision of the International Statistical Classification of Diseases and Related Health Problems (ICD-10), where each disease was assigned a code containing a letter and a number corresponding to its meaning. For bipolar affective disorder, it is F31, for depressive disorder―F32, recurrent depressive disorder―F33, and mixed anxiety and depressive disorder―F41.2 [6]. Paper copies of the questionnaires were distributed by a psychiatrist to the patients of the Department of Psychiatry during outpatient visits. The respondents were informed that participation in the study was anonymous and voluntary, and that they could opt out at any stage. Informed consent was obtained from all the subjects and/or their legal guardian(s). The patients, after consulting with a psychiatrist and agreeing to participate in the study, were given a paper version of the questionnaire, which they completed on their own. The completed questionnaires were placed in a box prepared for this purpose—for the sake of anonymity.

The author questionnaire consisted of two parts concerning sociodemographic data (age, sex, marital status, education, place of residence, employment status) and medical data (type of disorder, duration of the disease, medication time, number of medications used during the study period, attending medical appointments). The standardized research instruments were the Adherence to Refills and Medication Scale (ARMS), the Acceptance of Illness Scale (AIS), the Beck Depression Inventory (BDI), and the Satisfaction with Life Scale (SWLS).

The Adherence to Refills and Medication Scale (ARMS), developed by Kripalani et al., is a 12-item questionnaire consisting of two subscales: the first eight questions concern medication adherence, and the next four are about prescription adherence. Each answer is assigned a value: 1 = never, 2 = seldom, 3 = often, 4 = most of the time. The total score indicates the level of adherence—the lower the score, the better the adherence [27]. In the reliability analysis of the Polish version of the ARMS questionnaire, Cronbach’s standardized α was 0.954 [28].

The Acceptance of Illness Scale (AIS) by B. Felton, T. Revenson, and G. Hinrichsen was adapted to Polish conditions by Z. Juczyński. It comprises eight statements, each rated on a five-point Likert scale (from 1―I fully agree, denoting poor adaptation to a disease to 5―I fully disagree, meaning its full acceptance). The total score is a sum of all the points, and falls between 8 and 40. A low score reflects a lack of acceptance and poor adaptation to a disease, and high scores indicate a lack of negative emotions associated with the disease [29]. The Polish version of AIS has a high Cronbach α coefficient—0.82 [30].

The Satisfaction with Life Scale (SWLS) by E. Diener, R.A. Emmons, R.J. Larson, and S. Griffin measures the level of life satisfaction. It includes five items assessed on a seven-point Likert scale (from 1―I strongly disagree to 7―I strongly agree). The final score can range from 5 to 35 points—the higher the score, the higher the life satisfaction. The results should be interpreted with reference to the sten norms: 1–4 sten―low score, 5–6 sten―average score, 7–10 sten―high score [31]. Cronbach’s α for the Polish version of the Satisfaction with Life Scale (SWLS) developed by Zygfryd Juczynski is 0.81. This indicates good internal consistency of this tool in the Polish research context [30].

The Beck Depression Inventory (BDI) is one of the most widely used tools for assessing symptoms of depression [32,33]. The inventory contains 21 questions concerning sadness, the future, neglect, loss of pleasure in activities performed, feelings of guilt and punishment, the feeling of self-dislike, self-criticism, suicidal thoughts, tearfulness, nervousness, lack or loss of interests, indecisiveness, lower self-esteem, lack of energy, sleep rhythm, the feeling of fatigue, appetite, weight loss, and sexual interest [34]. The questions refer to the last 30 days of one’s life. Respondents can choose from four answer options with assigned values from 0 to 3 points, where 0 means no symptoms and 3 means significant severity. The higher the score, the more severe the depressive symptoms. The results are interpreted as follows: 0–11 points—no depressive symptoms, 12–26 points—mild symptoms, 27–49 points—moderate symptoms, 50–63 points—severe symptoms [33]. For the Polish version of the Beck Depression Inventory (BDI), Cronbach’s α coefficient is typically in the range of 0.89–0.92, depending on the sample. This indicates a very high internal consistency of the tool [34].

This study was approved by the Bioethics Committee of the Pomeranian Medical University in Szczecin (KB-0012/153/17; date 18 December 2017).

### Statistical Analysis

The quantitative and categorical variables (nominal and ordinal) were characterized using descriptive statistics methods. For the quantitative variables, the following were determined: measures of central tendency (M―mean, Mdn―median) and measures of variability (SD―standard deviation, IQR/2―interquartile range, CV―coefficient of variation). For the categorical variables, we determined the measures of the structure: number (n) and frequency (%). The results of the standardized psychometric measurements were calculated according to the rules described by their creators, and converted to a sten scale using norms for the Polish general population.

Classical statistics based on null hypothesis testing were used for statistical inference. The impact of the selected medical variables and the severity of the depressive symptoms on life satisfaction (the SWLS), adherence (the ARMS), and acceptance of the disease (the AIS) were analyzed using regression models. Multivariate linear regression models with least squares parameter estimation were tested. The multicollinearity of the independent variables was checked by determining the tolerance coefficient to avoid model redundancy.

The categorical variables were coded using a sigma-constraint method (quasi-experimental). All the predictors were entered into the model simultaneously. Stepwise progressive introduction of the predictors was used to build a determinant model that would explain the variance of the dependent variable to the greatest extent. The degree of the overall explained variance of the dependent variable was estimated by determining the adjusted R2 value. For each predictor, the unstandardized (b) and standardized (βstand.) regression coefficient was determined, along with the 95% confidence interval (95% CI).

A default level of statistical significance of 0.05 was assumed for all the analyses. Calculations were performed using the Statistica software v. 13.3 (TIBCO Software Inc., Palo Alto, CA, USA).

## 3. Results

### 3.1. Characteristics of the Study Sample

This study included 102 people with mood disorders. The study sample consisted of 91.18% women and 8.82% men, 64.71% of whom were in a relationship. Of the respondents, 57.84% had higher education, 14.71% had primary or vocational education, and 27.45% had secondary education. A total of 21.57% were rural residents, 30.39% lived in a city with a population of up to 100,000, and 48.04% were from a city with a population of over 100,000. The majority of the participants (59.8%) were employed compared to 40.2% of those being out of work. The mean age was 42.26 years, and the median was 38.5 years. The most numerous patients were those aged 20–25 years (32.9%).

As many as 47.06% of the respondents suffered from depressive disorder, 34.31% had mixed (anxiety–depressive) disorder, 11.76%―bipolar disorder, and 6.86%―other disorders.

The duration of the disorder ranged from one year to 36 years; the average time was 9.31 years, while the median was seven years. The most numerous group was the one in which the respondents had been ill for more than five years (more than 55%); 41.18% of the respondents had been ill for less than five years, and three people did not specify the duration of the disorder.

As for the time of taking medication, 85.29% of the participants took medication once a day in the morning, while 14.71% took medication at other times of the day. More than half of the respondents (53.92%) took two or three different drugs daily, 24.51% took one drug, and 21.57% took more than three drugs.

When it comes to medical appointments, 10.78% of the people deliberately did not attend a pre-arranged appointment.

### 3.2. Analysis of Depressiveness, Life Satisfaction Adherence, and Disease Acceptance Among Patients with Mood Disorders

An analysis of the severity of the depressive symptoms revealed that the majority of the respondents (45.10%) had moderate symptoms, 25.53% had mild depression, 18.63% had severe depression, and 12.75% had no depression.

Table 1 shows the descriptive statistics for each standardized questionnaire and its dimensions. The mean level of depression according to the BDI was 33.61, the median was 35, and the standard deviation was 17.24. The mean score for satisfaction with life was 15.8 and the median was 16, while the standard deviation was 6.65. The mean adherence level based on the ARMS was 19, the median was 18, and the standard deviation was 5.66. For the acceptance of the disease according to the AIS, the mean was 23.47, the median was 24, and the standard deviation was 7.97 (Table 1).

### 3.3. Analysis of the Impact of Treatment-Related Variables on Life Satisfaction, Adherence, and Disease Acceptance

An analysis of the effect of the treatment-related variables on life satisfaction according to the SWLS confirmed the influence of the number of medications used and the pattern of taking them throughout the day. The patients taking two or three medications had statistically significantly lower life satisfaction than those taking one medication (*SE* = −0.303, *p* = 0.005).

The patients taking medications more than once a day had statistically significantly lower satisfaction with life compared to their counterparts taking medications only once a day in the morning *(SE* = −0.213, *p* = 0.035). There was no effect of the type of disorder (bipolar affective disorder: *SE* = −0.103, *p* = 0.453; mixed disorders: *SE* = −0.231, *p* = 0.101), the duration of the disorder (*SE* = −0.085, *p* = 0.397), or the failure to attend medical appointments (*SE* = −0.105, *p* = 0.288) on the life satisfaction according to the SWLS (Table 2).

An analysis of the effect of treatment-related variables on the level of adherence confirmed the influence of the type of disorder and the conscious failure to attend a scheduled medical appointment in the past. The patients with bipolar disorder scored higher on the adherence scale (worse adherence to treatment recommendations) (*SE* = 0.308, *p* = 0.027), while the patients with mixed anxiety–depressive disorders had lower scores on the adherence scale (better adherence to treatment recommendations) than the patients with depression (*SE* = −0.364, *p* = 0.011). Those with a history of failing to attend a pre-arranged medical appointment scored higher on the adherence scale (worse adherence to treatment recommendations) compared to those without such experience (*SE* = 0.412, *p* = 0.490). Neither the duration of the disorder (*SE* = −0.069, *p* < 0.001) nor the number (two/three drugs: *SE* = 0.204, *p* = 0.055; more than 3 drugs: *SE* = −0.141, *p* = 0.197) or the frequency (multiple daily: *SE* = −0.007, *p* = 0.945) of the medications used had an effect on the adherence level as measured by the ARMS (Table 3).

There was no effect of medical data on the level of acceptance of the disease according to the AIS (Table 4).

### 3.4. Analysis of the Impact of Depressive Semiology on Life Satisfaction, Adherence, and Disease Acceptance

Then, we analyzed the impact of the severity of the depressive symptoms according to the BDI on life satisfaction according to the SWLS. We observed that the level of life satisfaction decreased with an increase in the severity of the depressive symptoms (*SE* = −0.665, *p* < 0.001). Mood disorder patients with more severe depressive symptoms had significantly higher scores on the adherence scale (worse adherence to treatment recommendations) (*SE* = 0.290, *p* = 0.003). The respondents with higher levels of depressive symptoms according to the BDI showed a lower level of acceptance of the disease according to the AIS (*SE* = −0.462, *p* < 0.001) (Table 5).

## 4. Discussion

Mood disorders, including symptoms related to anxiety and depression, are becoming increasingly common in the population [35,36,37]. Mood problems may be transient, but they affect people of all ages, sexes, social classes, and cultures. They occur worldwide, may begin early [38,39,40,41,42,43,44,45] and persist throughout life [46,47,48,49]. According to our study, people with bipolar disorder adhere to therapeutic recommendations worse, and those with mixed anxiety–depressive disorders adhere better than patients with depression.

### 4.1. The Influence of Adherence on Mood Disorders

When they studied the factors affecting the adherence to medical recommendations among psychiatric patients, De las Cuevas et al. noted that the adherence levels increased with trust in the treating physician. Non-adherence was estimated to occur in 13% to 56% of the patients taking antidepressants, and 20% to 60% of the patients with bipolar affective disorder [50]. According to Nierenberg et al. more than 50% of the patients with bipolar affective disorder did not adhere to the treatment recommendations [51], and in a study of 33,131 people conducted by Lintunen et al., the majority of the patients (59.1%) with bipolar disorder did not use their medications as prescribed [52]. Henein et al. [53], on the other hand, examined the adherence to the long-term use of the medications among 344 patients with clinically diagnosed depression, based on the pharmacy claims obtained. They found that the patients adhered to their medication regimen for about 85% of the days. Better adherence was associated with a lower likelihood of suicidal thoughts after one year of follow-up. Sawada et al. came to similar conclusions when they analyzed the persistence and adherence to antidepressant treatment in 367 patients with major depressive disorder. These authors monitored the adherence for six months, and noticed that only 161 (44.3%) of the patients continued antidepressant treatment for this period. Of the 252 patients who discontinued their antidepressant therapy, as many as 66.1% did so without first consulting their doctor. Based on a fairly high rate of treatment discontinuation without notifying the specialist, the authors concluded that special attention should be paid to closer communication between doctors and their patients [54]. Conti et al. suggest that lower adherence leads to a worse course of the disease and poorer quality of life [55]. In a study by Akincigil et al., conducted in a group of 4312 respondents with depression, the adherence rate in the 16th week of the study was 51%, and by the 33rd week it dropped to 21% [56]. Also, Melartin et al., who investigated persistence as a challenge in the treatment of major depressive disorder in psychiatric care, found that 88% of patients with depression received antidepressant treatment early in the acute phase, but almost half of them (49%) discontinued treatment prematurely. This decision was made under the influence of the worst course of major depressive episodes and a skeptical attitude resulting from the fear of antidepressant side effects [57]. Lingman and Scott maintain that a lack of adherence affects 10% to 60% of people with bipolar disorder [58].

The results of the study indicate varying levels of adherence in the study group, highlighting the need to pay attention to the factors affecting adherence to treatment recommendations. It is worth considering the role of systemic barriers, such as the availability of medicines and medical care, and psychosocial factors, including family and neighborhood support.

There is ample evidence of the beneficial effect of treatment adherence on the treatment process, but despite this knowledge, the adherence rates in people with mental disorders are low [50,51,52]. Patients may find it bothersome that despite taking medications they have to wait for visible effects, which is why it is so important not to give up on pharmacotherapy. This study suggests that adherence to medication can prevent suicidal thoughts in adults with severe depressive symptoms [53]. Patient education covering the essence of adherence is very important in the therapeutic process.

### 4.2. Factors Influencing Adherence Among Patients with Mood Disorders

Our analysis confirmed that people with mood disorders who reported a higher severity of depressive symptoms showed poorer adherence to treatment recommendations, highlighting the importance of considering their mental state when planning treatment. Also, De las Cuevaset et al., who investigated the risk factors for non-adherence to antidepressant treatment in patients with mood disorders in a group of 145 people, found that adherence significantly correlated with the severity of depression. They noted that patients with more severe depression were less compliant with medical recommendations. It is unclear whether the patients who were more depressed did not adhere to the recommendations due to a lack of willingness, or whether not taking medication led to increased depression [59]. Similar conclusions were formulated by Roca et al. [60] and Russel and Kazantzis [61], who observed worse adherence in the patients with more severe depressive symptoms. Rivero-Santana et al. observed an inverse cause-and-effect relationship between depression severity and medication adherence, suggesting that better adherence to treatment leads to better health [62].

It has also been found that the attitude of the main attending doctor plays an important role, and that appropriate communication between the doctor and the patient may have a positive impact on the adherence of the latter to treatment recommendations [58]. Similarly to ours, De las Cuevas et al.’s study of 966 psychiatric patients showed no effect on the type or the number of medications taken or the duration of the disease on adherence. They noted though that the adherence levels increased with trust in the treating physician. In contrast, when the patients were convinced of the rightness of their own actions regarding their mental health, they stopped following the specialist’s recommendations, and made decisions about treatment on their own [50].

Marasine and Sanchi analyzed the literature in search of the factors associated with non-adherence to antidepressant therapy among the patients with depression. They found that treatment non-adherence was strongly influenced by patient–doctor interactions. The most common problems included the doctor’s failure to provide sufficient information about the diagnosis and prescribed medications. The patients most often complained about the doctor’s unavailability and lack of time, incomplete information on the drug dosage and benefits of therapy, a lack of trust, and dissatisfaction with the treatment. All these aspects contributed to the discontinuation of treatment. Some patients changed or stop their treatment without informing their doctor, most often due to communication problems [63].

Also in our study, it was noted that the deliberate failure to attend a scheduled medical appointment had a negative impact on the compliance with treatment recommendations in a group of patients with mood disorders. Knowingly failing to attend a scheduled medical appointment is also associated with poorer adherence, suggesting that certain patient behaviors or attitudes may be an important risk signal for low adherence. In contrast, factors such as the duration of the illness, the number of medications used, or the frequency of taking the medications Did not appear to significantly affect adherence. Particular attention should be paid to the strengthening of the patient–physician relationship, as well as the correct communication between them. Doctors should place greater emphasis on patient education, which should include information about the drug being taken, the duration of therapy, and the preparation of the patient for the possible side effects and consequences of non-adherence.

Bull et al. examined the impact of patient–physician communication on discontinuing or making changes to antidepressant therapy among 401 patients treated for depression and 137 physicians who prescribed them medications. Of all the physicians, 72% said that they usually recommended taking medications for six months, while 21% did not inform the patients about the duration of treatment in order to monitor their well-being on an ongoing basis. As for the patients, 37% reported that their doctors had ordered the medication to be taken for six months, and 56% claimed that they had not received any instructions on the duration of therapy. Giving information about the adverse effects of taking medications significantly reduced the likelihood of discontinuing therapy, as did a follow-up visit to the doctor three or more times since the start of treatment [64]. Harmancı and Yıldız’s study has shown that psychoeducation and the use of motivational interviewing have positive effects on treatment adherence and functionality in people diagnosed with bipolar affective disorder [65]. The systematic review conducted by González de León et al. to assess the efficacy of the interventions improving medication adherence among adults with depressive disorders indicates that such interventions are effective for up to six months [66].

### 4.3. Life Satisfaction and Adherence Among Patients with Mood Disorders

In our investigation, people with a higher severity of depressive symptoms had lower life satisfaction, which may reflect the impact of worsened emotional state on subjective assessment of life satisfaction. Meule and Voderholzer also took life satisfaction into account in their study of 9649 people with mental disorders. According to these authors, the subjects with mental disorders reported lower levels of life satisfaction than the healthy controls. What is more, the level of satisfaction also differed between the diagnostic groups. They also noted that the life satisfaction increased during hospital treatment, which was indirectly related to the changes in the severity of the depressive symptoms, although, ultimately, the patients at discharge did not reach similar levels to the non-clinical sample [67]. In their two-year study on the costs of depression, Knorring et al. focused on the consequences of adhering to recommendations, response to treatment, and quality of life. The average quality of life score was initially low, but improved over the course of the study. The data indicated a more pronounced improvement in the quality of life among the patients who adhered to treatment recommendations compared to those who did not [68]. Dzevlan et al. studied the effect of taking antidepressants on improvement in the quality of life of 682 adult patients diagnosed with depression and/or anxiety disorders. An analysis of the data showed that adherence to the prescribed medication regimen improved the quality of life and life satisfaction in the patients with depression, anxiety, and mixed disorders [69]. Ramirez and Carver-Enguix demonstrated the impact of depression severity on the quality of life of the depressed patients [70]. Investigating the effects of remission and somatic symptoms on the quality of life and life satisfaction of patients with major depressive disorder, Woo et al. proved that the life satisfaction was significantly higher in the patients without remission than in those with remission [71]. In Herman et al.’s study, the patients who reported more severe depressive symptoms were less satisfied with their health status [72]. Another study comparing the quality of life of 165 depressed patients to that of 165 healthy controls found that depression impaired functioning in everyday life and had a significant impact on satisfaction with health, mental and physical well-being, and bodily pain [73].

Sagy et al. described a positive correlation between medical visits and the severity of depression. They also found a positive correlation between depression and the number of medications taken [74].

In a study of 157 patients, focusing on subjective life satisfaction and objective functional outcomes in bipolar and unipolar affective disorders, Goldberg and Harrow proved that recurrent depression substantially contributed to the deterioration of life satisfaction in patients with bipolar disorder [75], which was also observed in our study. The solution to the above problems of the patients with bipolar affective disorder may be psychoeducation and participation in psychotherapeutic (cognitive–behavioral) classes, where they can obtain support from other people suffering from the same disease. Javadpour [76] and Costa [77] confirmed the positive impact of the above therapies on the overall healing process—the individuals participating in such meetings had greater knowledge of the symptoms, course, and consequences of the disease, and their quality of life improved in all areas compared to those who did not use the aforementioned options.

### 4.4. Implications

Non-adherence to treatment recommendations is a common phenomenon among patients with mood disorders. It is a problem that has negative health and social consequences. A key element that can affect adherence in patients with mood disorders is education about the nature of long-term medication use. Care should be taken to ensure that patients understand how pharmacotherapy works and why they should not discontinue it despite the initial lack of visible effects. Frequent consultations during which educational activities would be conducted could increase patient engagement in treatment and lead to improved therapeutic outcomes. In addition, increasing trust between the patient and the doctor through regular communication and showing concern for the patient can help reduce the feelings of uncertainty and frustration that often lead to treatment discontinuation. In addition to pharmacotherapy, it is useful to offer patients with psycho-logical support, such as through cognitive–behavioral therapy, which can help them cope with the difficulties associated with the disease and better accept treatment. A holistic approach will support the patient at every stage of treatment and increase his or her motivation to adhere to treatment. It is also important to promote public awareness of mood disorders to reduce patient stigma. Increased public awareness of mood disorders will benefit patients’ acceptance of the disease and their commitment to treatment.

### 4.5. Limitations

One of the main limitations of this study is the relatively small sample size—102 participants. In order to fully justify the selection of this sample size, it would be useful to conduct a power analysis to determine whether the sample was sufficient to detect significant statistical effects. A lack of power analysis can undermine the reliability of the results, especially in the context of small samples, where the risk of Type I or II error is greater.

Another limitation is the unequal gender representation in the sample—91.18% women—which may have limited the generalizability of the results to the entire population. Although women are more likely to suffer from mood disorders, this disproportion in the sample may make it difficult to draw conclusions about gender differences in health behavior, the adherence to treatment recommendations, or the impact of illness on quality of life. Future studies should include a more balanced gender representation to better reflect the epidemiology of mood disorders in the population.

Additionally, this study included few patients with bipolar disorder (11.76%), which may limit the ability to draw conclusions about this specific subtype of mood disorder. Bipolar affective disorder varies in terms of symptoms, treatment, and impact on a patient’s life, so results on general mood disorders may not be fully representative of this group. For a more complete picture, future studies should include a larger sample of patients with this disease entity.

The study period was from December 2020 to January 2022, which coincided with the ongoing COVID-19 pandemic. External factors related to the pandemic may have affected the adherence to treatment and the severity of the mood disorder symptoms. Social isolation, uncertainty, stress, and the reduced availability of health services may have significantly affected the patients’ behavior, mood and coping. This should be taken into account as a potential limitation of this study, as the results may be incomplete or distorted in the context of the global health crisis.

## 5. Conclusions

1. The number of medications taken, and the severity of the depressive symptoms determine the life satisfaction of people with mood disorders;
2. The respondents with a greater severity of depressive symptoms scored higher on the adherence scale, which means that they were more likely to be non-adherent to the treatment recommendations. The type of mood disorder may affect patient adherence. The subjects with bipolar disorder showed higher adherence and those with anxiety–depressive disorder showed lower adherence than the patients with depression;
3. The subjects with more severe depressive symptoms showed a lower degree of acceptance of the disease.

## Figures and Tables

**Table 1:** Depressive symptoms according to the BDI; satisfaction with life—the SWLS; adherence in chronic diseases—the ARMS; and acceptance of the disease—the AIS.

Variable	Mean	SD	Median	IQR/2	Minimum	Maximum	CV (%)
BDI	33.61	17.24	35.0	12.50	0.0	70.0	51.3
SWLS	15.80	6.65	16.0	5.50	5.0	34.0	42.1
ARMS	19.13	5.66	18.0	4.00	12.0	35.0	29.6
AIS	23.47	7.97	24.0	6.50	8.0	40.0	34.0

SD—standard deviation; IQR/2—interquartile range; CV—coefficient of variation.

**Table 2:** The influence of treatment-related variables on the level of life satisfaction according to the SWLS.

Variable		Estimate	*SE*	−95% CI	+95% CI	*p*
Intercept		16.372				<0.001
Type of disorder	depression (ref.)					
	bipolar affective disorder	−0.992	−0.103	−0.376	0.169	0.453
	mixed disorders	−1.682	−0.231	−0.509	0.046	0.101
Duration of the disorder	up to 5 years (ref.)					
	over 5 years	−0.577	−0.085	−0.283	0.113	0.397
Number of medications used	one (ref.)					
	two/three	−2.539	−0.303	−0.511	−0.095	0.005
	more than three	1.519	0.147	−0.068	0.362	0.177
Taking medications	once in the morning (ref.)					
	several times	−1.993	−0.213	−0.410	−0.016	0.035
Failure to attend medical appointments		−1.132	−0.105	−0.299	0.090	0.288

*SE*—standard error of estimate; CI—confidence interval ref.—reference level.

**Table 3:** The influence of treatment-related variables on the level of adherence according to the ARMS.

Variable		Estimate	*SE*	−95% CI	+95% CI	*p*
Intercept		22.361				<0.001
Type of disorder	depression (ref.)					
	bipolar affective disorder	2.508	0.308	0.035	0.581	0.027
	mixed disorders	−2.245	−0.364	−0.643	−0.086	0.011
Duration of the disorder	up to 5 years (ref.)					
	over 5 years	−0.398	−0.069	−0.268	0.129	0.490
Number of medications used	one (ref.)					
	two/three	1.449	0.204	−0.004	0.413	0.055
	more than three	−1.233	−0.141	−0.356	0.075	0.197
Taking medications	once in the morning (ref.)					
	several times	−0.055	−0.007	−0.205	0.191	0.945
Failure to attend medical appointments		3.773	0.412	0.217	0.607	<0.001

*SE*—standard error of estimate; CI—confidence interval; ref.—reference level.

**Table 4:** The influence of medical variables on acceptance of the disease according to the AIS.

Variable		Estimate	*SE*	−95% CI	+95% CI	*p*
Intercept		24.235				<0.001
Type of disorder	depression (ref.)					
	bipolar affective disorder	−2.702	−0.236	−0.532	0.061	0.118
	mixed disorders	0.266	0.031	−0.271	0.333	0.840
Duration of the disorder	up to 5 years (ref.)					
	over 5 years	0.007	0.001	−0.215	0.217	0.994
Number of medications used	one (ref.)					
	two/three	−0.966	−0.097	−0.323	0.130	0.398
	more than three	0.793	0.064	−0.170	0.298	0.586
Taking medications	once in the morning (ref.)					
	several times	−2.223	−0.199	−0.414	0.016	0.069
Failure to attend medical appointments		−0.044	−0.003	−0.215	0.209	0.975

*SE*—standard error of estimate; CI—confidence interval; ref.—reference level.

**Table 5:** The influence of depressive symptoms according to the BDI on life satisfaction according to the SWLS, the adherence in chronic diseases according to the ARMS, and the acceptance of the disease according to the AIS.

Variable	Estimate	*SE*	−95% CI	+95% CI	*p*
Intercept	24.459				<0.001
BDI	−0.258	−0.665	−0.813	−0.517	<0.001
Intercept	15.825				<0.001
ARMS	0.095	0.290	0.100	0.480	0.003
Intercept	30.752				<0.001
AIS	−0.215	−0.462	−0.638	−0.286	<0.001

*SE*—standard error of estimate; CI—confidence interval; ref.—reference level.

## Data Availability

The dataset is available upon request from the corresponding author at the email address: daria.schneider.matyka@pum.edu.pl.
